# Altered Expression of* EPO* Might Underlie Hepatic Hemangiomas in* LRRK2* Knockout Mice

**DOI:** 10.1155/2016/7681259

**Published:** 2016-10-30

**Authors:** Ben Wu, Kaifu Xiao, Zhuohua Zhang, Long Ma

**Affiliations:** The State Key Laboratory of Medical Genetics, School of Life Sciences, Central South University, No. 110 Xiangya Road, Changsha, Hunan 410078, China

## Abstract

Parkinson's disease (PD) is a severe neurodegenerative disorder caused by progressive loss of dopaminergic neurons in the substantia nigra pars compacta of the midbrain. The molecular mechanism of PD pathogenesis is unclear. Mutations in the* leucine-rich repeat kinase 2* (*LRRK2*) gene are a common genetic cause of familial and sporadic PD. However, studies on* LRRK2* mutant mice revealed no visible dopaminergic neuronal loss in the midbrain. While surveying a* LRRK2* knockout mouse strain, we found that old animals developed age-dependent hepatic vascular growths similar to cavernous hemangiomas. In livers of these hemangioma-positive* LRRK2 *knockout mice, we detected an increased expression of the HIF-2*α* protein and significant reactivation of the expression of the HIF-2*α* target gene* erythropoietin* (*EPO*), a finding consistent with a role of the HIF-2*α* pathway in blood vessel vascularization. We also found that the kidney* EPO* expression was reduced to 20% of the wild-type level in 18-month-old* LRRK2* knockout mice. Unexpectedly, this reduction was restored to wild-type levels when the knockout mice were 22 months to 23 months old, implying a feedback mechanism regulating kidney* EPO* expression. Our findings reveal a novel function of* LRRK2* in regulating* EPO* expression and imply a potentially novel relationship between PD genes and hematopoiesis.

## 1. Introduction

Parkinson's disease (PD) is the second most common age-related neurodegenerative disorder and the most common movement disorder [1]. PD patients usually exhibit clinical features such as resting tremor, bradykinesia, rigidity, and postural instability as a result of the progressive loss of the dopaminergic neurons located within the substantia nigra pars compacta (SNpc) of the midbrain. Visible loss of the SNpc dopaminergic neurons and subsequent development of dystrophic striatal projections are the hallmarks of PD [1].

Dopaminergic neurons of PD patients normally contain Lewy bodies and Lewy neurites, intracellular protein aggregates formed primarily by the *α*-synuclein protein [1]. Mutations in* SNCA (a-synuclein)* and* LRRK2* are the major causes of autosomal dominant forms of PD [1–4]. Loss-of-function mutations in other genes, for example,* Parkin*,* PINK1*, and* DJ-1*, are the causes of autosomal recessive forms of PD [1, 5–7].

*LRRK2* (*PARK8*) was initially identified in autosomal dominant familial PD patients [3, 4]. Subsequent studies identified* LRRK2* mutations to be the major cause of familial and sporadic PD [8].* LRRK2* encodes a 2527-amino acid protein with both kinase and GTPase activities and in human is expressed in multiple tissues [3, 4]. Studies of* LRRK2* mutant mice (knockins and knockouts) suggest that* LRRK2* might be involved in neural process outgrowth, synaptic vesicle dynamics, and the autophagy/lysosomal pathway [9–15]. However, no apparent loss of dopaminergic neurons was detected in these different* LRRK2* mutant mice.

Recently, Tong et al. found that the kidneys of a* LRRK2* knockout mouse strain exhibit profound *α*-synuclein aggregation, oxidative stress, inflammation, and cell death [14].* LRRK2* knockout mice also have early-onset increase in the number and size of secondary lysosomes in kidney proximal tubule cells and lamellar bodies in lung type II cells [16].* LRRK2* knockout rats were found to have abnormal morphologies and/or functions in kidney, spleens, lung, and liver [9, 10]. These findings suggest that* LRRK2* might have important functions in peripheral organs.

We postulate that phenotypes in peripheral organs of* LRRK2* mutant mice might be more obvious in aged animals, similar to the age-dependent degeneration of dopaminergic neurons in PD patients, and these phenotypes could serve as readouts for understanding the molecular functions of* LRRK2*. By scrutinizing peripheral organs of a* LRRK2* knockout mouse line [14], we found that these mice develop age-dependent hepatic growths similar to hemangiomas. We also detected altered expression of* EPO* in the livers and kidneys of the knockout mice. Our findings suggest a previously unknown relationship between* LRRK2* and hematopoiesis.

## 2. Materials and Methods

### 2.1. *LRRK2* KO Mice

The* LRRK2* KO (*LRRK2* ^−*/*−^) mice (stock number 016209) [14] were purchased from the Jackson Laboratory (Maine, USA). Genotyping was performed as described [14]. F_1_ wild-type or* LRRK2* ^−*/*−^ homozygous mice of the B6/129 genetic background were obtained by crossing P_0_ *LRRK2* ^−*/*+^ heterozygous mice. Littermate F_1_ wild-type mice were crossed to generate F_2_ wild-type controls and littermate F_1_ *LRRK2* ^−*/*−^ homozygous were crossed to generate F_2_ *LRRK2* ^−*/*−^ mice. Analysis was performed with F_2_ mice. This study was conducted in compliance with the regulations of the Institutional Animal Care and Use Committee of the State Key Laboratory of Medical Genetics of CSU in China.

### 2.2. Quantitative RT-PCR

Total RNA was extracted from kidney and liver using TRIzol (Invitrogen) and treated with RNase-Free DNase I (New England Biolabs). First-strand cDNA was synthesized using the Superscript III First-Strand Synthesis Kit (Thermo Scientific). ~1 *μ*g total RNA from each sample was used for RT experiments. qPCRs were performed in triplicate for each sample using the Maxima SYBR Green qPCR Master Mix (Thermo Scientific). Fluorescence signals were detected using Bio-Rad CFX96 real-time cycler with the following cycling conditions: 10 min at 95°C, followed by 40 two-step cycles of 15 s at 95°C, and 20 s at 60°C.* GAPDH* is the reference gene. The expression level of each gene was quantified using Bio-Rad CFX Manager 3.0 and ΔΔCt method. PCR primers are listed in supplementary Table S2 in Supplementary Material available online at http://dx.doi.org/10.1155/2016/7681259.

### 2.3. Western Blot

Kidneys and livers were homogenized in homogenization buffer (10 v/w) containing 50 mM Tris (pH 7.4), 150 mM NaCl, 1% Triton X-100, 1% sodium deoxycholate, 0.1% SDS, 1 mM sodium orthovanadate, and 25 mM sodium fluoride, supplemented with protease inhibitor mixtures (Thermo Scientific) and phosphatase inhibitor mixtures (Thermo Scientific). 20 *μ*g proteins were loaded onto 10% SDS-polyacrylamide gels. Following electrophoresis, proteins were transferred to an Immobilon -P membrane (Millipore, USA). Membranes were blocked for 1 hour with 5% nonfat milk in Tris-buffered saline containing 0.1% Tween 20 (TBST). Membranes were probed with the primary antibodies diluted in 5% BSA in TBST. The dilution for each antibody was as follows: rabbit anti-LRRK2 mAb (Epitomics), 1 : 3000; rabbit anti-GAPDH pAb (Santa Cruz), 1 : 2000; and rabbit anti-HIF-2*α* mAb (Santa Cruz), 1 : 2000. After washing with TBST, the blots were incubated with secondary antibodies (Jackson ImmunoResearch) conjugated to horseradish peroxidase. Immunoreactive bands were detected by the chemiluminescence reagent (ECL) (GE Healthcare, UK).

### 2.4. Histological and Immunohistochemical Analysis

Mice were anesthetized by intraperitoneal injection of 10% chloral hydrate and perfused with 0.9% saline. The kidneys and livers were dissected out and postfixed in 4% paraformaldehyde in PBS (pH 7.4) at 4°C and then processed for paraffin embedding. Kidneys and livers were cut as 5 *μ*m thick sections on a microtome (Leica). For histopathological analysis, tissue sections were stained with hematoxylin and eosin (H&E). For immunohistochemical analysis, tissue sections were subjected to antigen retrieval by microwaving for 15 min in 10 mM sodium citrate buffer, pH 6.0. Endogenous peroxidase activity was quenched by incubating in 3% H_2_O_2_. After blocking, sections were incubated with primary antibodies overnight at 4°C followed by 30 min incubation with HRP-conjugated secondary antibodies (ZSGB-BIO, Beijing, China) and then developed using chromogenic DAB substrate (ZSGB-BIO, Beijing, China).

### 2.5. Hematological Examination

Hematological parameters were evaluated on animals of different ages and analyzed on Beckman Coulter Ac.T 5diff AL automated hematology analyzer (Beckman Coulter, USA). Blood samples were collected via the submandibular vein. Potassium EDTA was used as anticoagulants.

### 2.6. Statistical Analysis

Statistical analysis was performed using Prism 5 (GraphPad Software) and Excel (Microsoft). Data are presented as means ± SEM. Statistical significance was determined by *P* values of Student's *t*-test.

## 3. Results

### 3.1. *LRRK2* Knockout Mice Had Hepatic Hemangiomas

The* LRRK2* knockout (*LRRK2* ^−*/*−^) mice had grossly normal dopaminergic neurons at 2 years of age [14]. Interestingly, these* LRRK2* ^−*/*−^ mice exhibit age-dependent renal atrophy, increased *α*-synuclein aggregation, impaired autophagy-lysosome pathway, and increased apoptosis and inflammation in kidneys [14].

**Figure 1:**
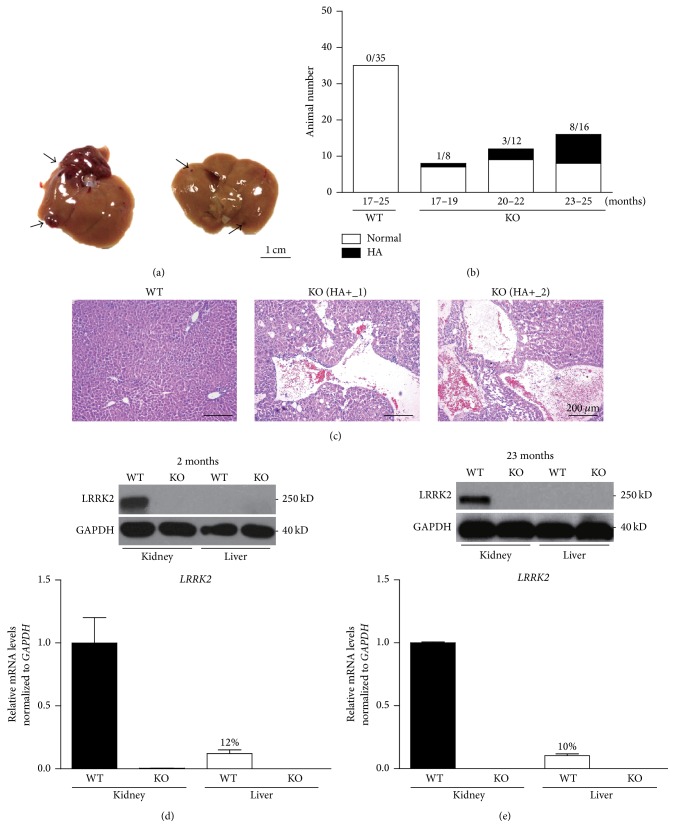
Hepatic hemangiomas in* LRRK2* mutant mice. (a) Gross morphology of a large hemangioma (left) and a small hemangioma (right). (b) Incidence of hepatic hemangiomas detected in* LRRK2* ^−*/*−^ mice at different ages. (c) Histological staining of WT control (left, 23 months of age) and* LRRK2* ^−*/*−^ (middle and right, 23 and 25 months of age, resp.) mutant liver sections. Large vascular spaces filled with blood cell were observed in* LRRK2* ^−*/*−^ mice. Scale bars: 200 *μ*m. (d, e) Western blotting indicates that no obvious LRRK2 expression is detected in the livers at ages of 2 months ((d), upper panel) and 23 months ((e), upper panel). Quantitative RT-PCR showing relative expression levels of* LRRK2* mRNAs in the kidneys and livers of WT and KO mice (*n* = 3) at the ages of 2 months ((d), lower panel) and 23 months ((e), lower panel). All data are expressed as mean ± SEM.

We examined other peripheral organs of old* LRRK2* ^−*/*−^ mice and found that a proportion of these mice exhibited hepatic growths that resembled vascularized blood vessels (Figure 1(a)). 12 of 36* LRRK2* ^−*/*−^ mice (33%) had such growths (Figure 1(b)), the youngest of which was 19 months old. No such growths were found in 35 wild-type controls. The sizes of the growths vary from those obvious with naked eyes (Figure 1(a), left) to those of microscopic scales (Figure 1(a), right). Among the 12 mice with the growths, five were males and seven were females, suggesting that sex might not affect the phenotype.

We examined liver sections of the* LRRK2* ^−*/*−^ mice and found that the growths contained tangles of small and large blood vessels partially or completely filled with red blood cells (Figure 1(c)). This phenotype was not observed in the livers of control mice (Figure 1(c), left) or* LRRK2* ^−*/*−^ mice without the liver growths (BW and LM, unpublished observations). The gross morphology and the microscopic structure of the liver growths are similar to the characteristics of hepatic hemangiomas [17], a benign tumor composed of hepatic endothelial cells [18].

In humans* LRRK2* is expressed in multiple tissues including the brain, heart, lung, kidney, and liver [3, 4]. In mice* LRRK2* is abundantly expressed in the brain, lung, heart, kidney, spleen, and lymph node and weakly in liver, eye, skeletal muscle, smooth muscle, thymus, stomach, and small intestine [19, 20]. We examined LRRK2 expression in the kidneys and livers at two different ages using Western blot (Figures 1(d) and 1(e), upper panels). As expected, LRRK2 is abundantly expressed in the kidneys and the* LRRK2* ^−*/*−^ mutation completely abolishes the expression (Figures 1(d) and 1(e)). However, no obvious LRRK2 expression was detected in the livers by Western blot. We further examined* LRRK2* mRNA expression by qRT-PCR (Figures 1(d) and 1(e), lower panels). Here we detected a residual* LRRK2* expression (12% and 10%, resp.) in the livers at both ages, consistent with the weak expression of* LRRK2* reported previously [19, 20].

### 3.2. Increased* Erythropoietin* Expression in the Livers of HA-Positive* LRRK2* ^−*/*−^ Mice

The genetic cause of hepatic hemangiomas is unclear. Previous studies found that the signal pathway involving the von Hippel-Lindau (VHL) factor, the HIF-2*α* transcriptional factor, and HIF-2*α* target gene* erythropoietin* (*EPO*) could affect the formation of cavernous hemangiomas in mouse livers [17, 21].

**Figure 2:**
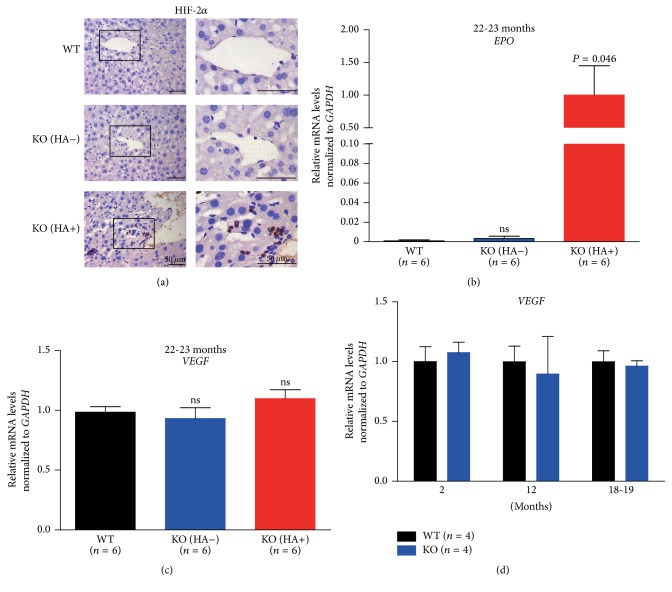
Increased* erythropoietin* (*EPO*) expression in the livers of HA-positive* LRRK2* ^−*/*−^ mice. Immunohistochemical analysis of HIF-2*α* expression in the livers of HA-positive* LRRK2* ^−*/*−^ mice at the age of 22-23 months. Nuclear HIF-2*α* staining was detected in clusters of cells near the vascularized vessels. Scale bars: 50 *μ*m. qRT-PCR analysis of* EPO* (b) and* VEGF* (c) mRNA levels in the livers of 22-23-month-old mice. (d) qRT-PCR analysis of* VEGF* mRNA levels in the livers of mice at ages of 2, 12, and 18-19 months. ns, not significant.

We examined HIF-2*α* expression by immunohistochemistry and found an apparent increase of HIF-2*α* signal in the nuclei of numerous cells surrounding the vascularized vessels in the livers of hemangioma- (HA-) positive* LRRK2* ^−*/*−^ mice (Figure 2(a), HA+). However, no visible HIF-2*α* signal was detected in livers of wild-type or HA-negative* LRRK2* ^−*/*−^ mice (Figure 2(a), WT and HA−, resp.). Similarly, no visible HIF-2*α* signals were detected in the livers of either wild-type or* LRRK2* ^−*/*−^ mice of younger ages (supplementary Figure S1).

To determine whether the increased expression of HIF-2*α* has any consequences, we examined the transcript level of its target gene* erythropoietin* [21] by qRT-PCR. In HA-positive* LRRK2* ^−*/*−^ mice, we detected an apparently increased (reactivated)* EPO* expression (Figure 2(b), HA+), which was not detected or was barely detected in the livers of either wild-type or HA-negative* LRRK2* ^−*/*−^ mice (Figure 2(b)). In addition, no obvious* EPO* signals were detected in the livers of both wild-type and* LRRK2* ^−*/*−^ mice of younger ages (BW and LM, unpublished observations).

To identify other factors that might be involved in the formation of hemangiomas in* LRRK2* ^−*/*−^ mice, we examined the transcript level of the* vascular endothelial growth factor (VEGF)* gene [22]. Similar levels of* VEGF* were detected in the livers of 22-month-old to 23-month-old mice of both wild-type and* LRRK2* ^−*/*−^ genotypes (Figure 2(c)). The presence of hepatic hemangiomas did not alter the expression either (Figure 2(c)). This similarity was also found in wild-type and* LRRK2* ^−*/*−^ mice of younger ages (Figure 2(d)). Therefore,* LRRK2* might not affect the expression of* VEGF* in the liver.

### 3.3. Altered* EPO* Expression in the Kidneys of* LRRK2* ^−*/*−^ Mice

During embryonic development, the liver is the major site of EPO production, which is taken over by the kidney after birth [21]. It is possible that the altered expression of* EPO* in the livers of* LRRK2* ^−*/*−^ mice might also be related to a change of* EPO* expression in the kidneys.

**Figure 3:**
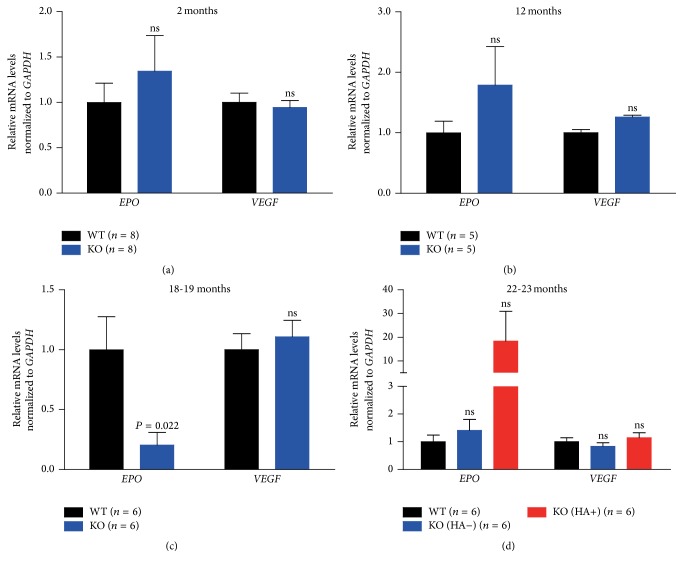
Expression of* EPO* and* VEGF* in the kidneys of mice at different ages. qRT-PCR analysis of* EPO* and* VEGF* mRNA levels in the kidneys of* LRRK2* ^−*/*−^ mice at the ages of 2 (a), 12 (b), 18-19 (c), and 22-23 (d) months.* EPO* expression in the kidneys of 18-19-month-old* LRRK2* ^−*/*−^ mice was reduced to ~20% of the wild-type level, which was restored to wild-type levels at the age of 22-23 months. ns, not significant.

We measured* EPO* expression in the kidneys of mice of different ages. At the ages of 2 and 12 months, the expression was similar between wild-type and* LRRK2* ^−*/*−^ mice (Figures 3(a) and 3(b)). However, in the kidneys of 18-month-old to 19-month-old* LRRK2* ^−*/*−^ mice,* EPO* expression was reduced to ~20% of the wild-type level (Figure 3(c)). This reduction was restored in* LRRK2* ^−*/*−^ mice at 22 to 23 months of ages (Figure 3(d)). We found that five of the six HA-positive* LRRK2* ^−*/*−^ mice had apparently increased* EPO* levels compared to wild-type mice (supplementary Table S1). However, no significant difference was detected between these groups by statistics probably due to the wide variations of the values for the HA-positive group (Figure 3(d) and supplementary Table S1).

Similar to the results in the livers, we did not detect an obvious difference in* VEGF* expression in the kidneys of wild-type and* LRRK2* ^−*/*−^ mice at different ages (Figures 3(a), 3(b), 3(c), and 3(d)).

We tried but failed to detect an apparent HIF-2*α* signal in the kidneys of both wild-type and* LRRK2* ^−*/*−^ mice at different ages by Western blot (BW and LM, unpublished observations) or immunohistochemistry (supplementary Figures S2A and S2B). In the kidneys of 22-23-month-old* LRRK2* ^−*/*−^ mice, we observed apparent histochemical signals that were not caused by HIF-2*α* immunostaining (supplementary Figure S2A). These signals might be similar to the increased autolysosome and lipofuscin signals described previously [11].

**Figure 4:**
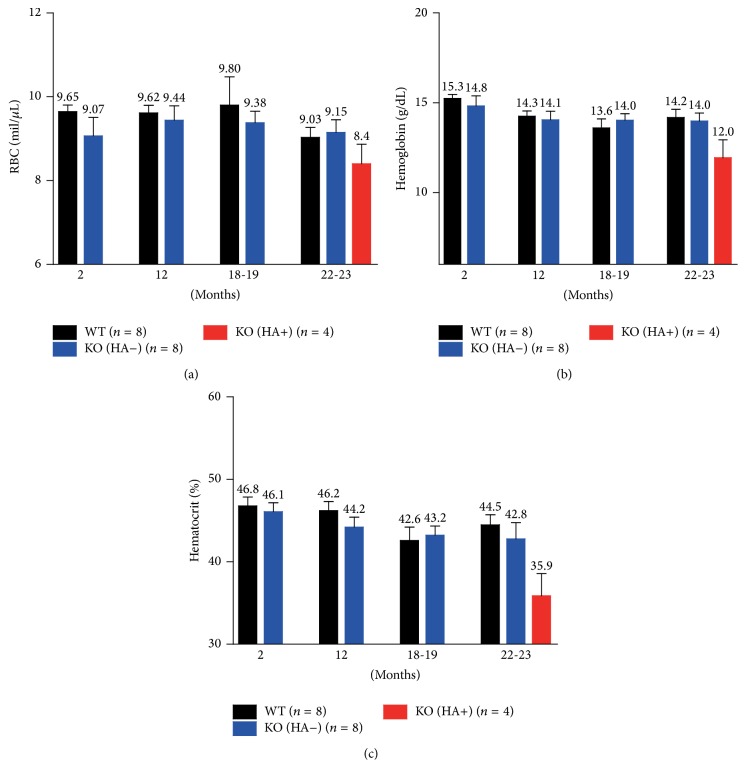
Hematological analysis of* LRRK2* ^−*/*−^ mice at different ages. Red blood cell (RBC) counts (a), hemoglobin concentration (b), and hematocrit value (c) in mice at the ages of 2, 12, 18-19, and 22-23 months. No significant difference in these parameters can be detected in all groups at different ages.

Since an altered* EPO* expression was detected in the* LRRK2* ^−*/*−^ mice, we examined whether this change would be linked to any alterations in the hematological profiles of these mice. In all groups at different ages, we failed to detect a significant difference in red blood cell count (RBC count), hemoglobin concentration, and hematocrit value (Figures 4(a), 4(b), and 4(c)). Therefore, a more extensive study is warranted to determine whether any* EPO*-related processes are altered in these mice.

## 4. Discussion

In this study, we report the identification of hemangioma-like growths in the livers of old* LRRK2* ^−*/*−^ mice and found that* LRRK2* could affect the expression of* EPO* in the livers and kidneys of these mice.

*LRRK2* mutations are the most frequent genetic causes of familial and sporadic PD [8]. To date, no apparent dopaminergic neuron degeneration was observed in various* LRRK2* mutant mice [13–16], raising the question that unknown genetic differences might be responsible for the discrepancy between human and mouse. Interestingly,* LRRK2* mutant rodents, including mice and rats, had physiological defects in peripheral organs such as kidney, lung, and liver [9–11, 13, 14, 16]. Considering the current lack of understanding on PD-related genes, a detailed analysis of* LRRK2* in peripheral organs not only is meaningful but also could provide novel insights into PD pathogenesis.

We found that 33% of* LRRK2* ^−*/*−^ mice after the age of 19 months develop liver growths that are similar to hepatic hemangiomas, suggesting that* LRRK2* might have an age-dependent function in affecting either erythropoiesis or angiogenesis in mice. The incomplete penetrance of this phenotype implies that either unidentified genetic modifiers or environmental factors are involved.

The genetic cause of hepatic hemangiomas remains unclear. Loss-of-function mutations in the* von Hippel-Lindau* tumor suppressor gene (*VHL*) could lead to age-dependent hepatic hemangiomas in mice [17]. Inactivation of HIF-2*α* can suppress the formation of hepatic hemangiomas in* VHL* mutant mice [23], suggesting that HIF-2*α* might promote the formation of hepatic hemangiomas. Consistent with this notion, we found that cells lining the vascularized vessels in the livers of HA-positive* LRRK2* ^−*/*−^ mice exhibited apparently increased expression of HIF-2*α*. This increase was not found in the livers of wild-type and HA-negative* LRRK2* ^−*/*−^ mice. Similarly, the expression of the HIF-2*α* target gene* EPO* was dramatically increased (reactivated) in the livers of HA-positive* LRRK2* ^−*/*−^ mice, suggesting a close correlation of* EPO* expression and the formation of hemangiomas in* LRRK2* ^−*/*−^ mice. Furthermore, the implication of* LRRK2* in this process provides a new insight into the genetics of hepatic hemangiomas.

*VEGF* is a signal protein that promotes vasculogenesis and angiogenesis [24]. We measured* VEGF* expression in mice at four different ages (2, 12, 18-19, and 22-23 months) but failed to detect an apparent change in either the kidneys or livers of* LRRK2* ^−*/*−^ mice with or without hepatic hemangiomas. Hence, the altered expression of* EPO* but not* VEGF* in* LRRK2* ^−*/*−^ mice suggests a special relationship between* LRRK2* and* EPO*, which does not simply result from a general defect caused by the* LRRK2* mutation.

In embryos, the liver is the major site for EPO production, while in adults the kidney takes over this role [21]. We found that* EPO* expression is significantly reduced in the kidneys of 18-19-month-old* LRRK2* ^−*/*−^ mice. Interestingly, the reduction was restored when the mice became 22 to 23 months old, implying a feedback mechanism. The reduced* EPO* expression might be caused by impaired kidney functions, since these mice exhibited renal atrophies by the age of 7 months and severe renal injuries at 20 months of age [11, 14]. Alternatively, this change might suggest a regulatory role of* LRRK2* in the expression of* EPO*. Future studies might reveal the underlying mechanism that can distinguish these two possibilities.

Our findings provide a first line of genetic evidence that* LRRK2* might be intrinsically related to* EPO* expression. Following the line, other PD genes might also be involved in* EPO* expression in adult kidneys. Indeed, a recent epidemiological study found that PD patients tend to have anemia years before developing PD symptoms [25]. Our findings also provide supporting evidence for the notion that the neural protective function of EPO could be applied for treating PD and other neurodegenerative diseases [26–28].

In short, we found that* LRRK2* could affect* EPO* expression in the kidneys and livers of aged mice, which might be related to the formation of hepatic hemangiomas. Future investigation into this phenomenon might provide new molecular details into the function of* LRRK2* in peripheral organs and PD pathogenesis.

## Supplementary Material

HIF-2a immunohistochemistry, EPO levels in individual mouse and list of PCR primers.
