# Supplementary material for: Altered Expression of EPO Might Underlie Hepatic Hemangiomas in LRRK2 Knockout Mice
Source: Biomed Res Int. 2016 Oct 30;2016:7681259. doi: 10.1155/2016/7681259 (PMC5107217; doi:10.1155/2016/7681259)
Supplement: Supplementary file 1 — HIF-2a immunohistochemistry, EPO levels in individual mouse and list of PCR primers. [file 7681259.f1.pdf]

Supplementary Figure S1. HIF-2α expression in the livers of mice at the ages of 2, 12 and 18-19 months

No visible HIF-2α signals can be detected in the livers of wildtype or *LRRK2*$^{-/-}$ mice at these ages. Scale bars: 50 μm.

Supplementary Figure S2. HIF-2α expression in the kidneys of mice at the ages of 2, 12 18-19 and 22-23 months

(A) Non-specific HIF-2α signals can be detected in the kidneys of 22-23 months old *LRRK2*$^{-/-}$ mice. (B) No apparent HIF-2α signals can be detected in the kidneys of wildtype or *LRRK2*$^{-/-}$ mice at the ages of 2, 12 and 18 months. Scale bars: 50 μm.

Figure S1

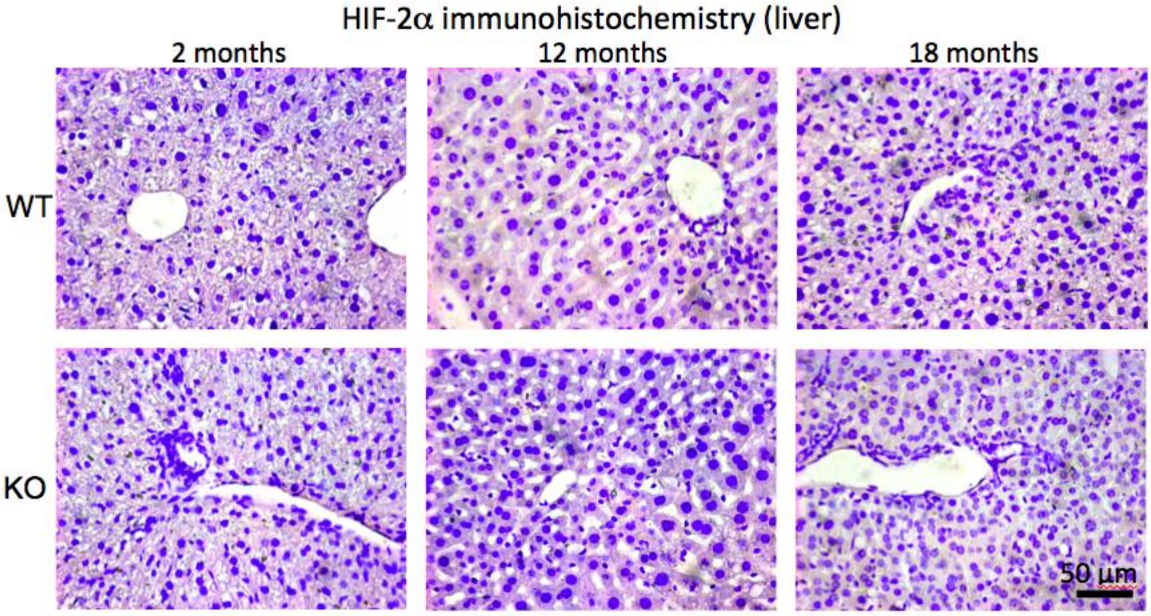
HIF-2α immunohistochemistry (liver)
2 months
12 months
18 months
WT
KO
50 µm

Figure S2

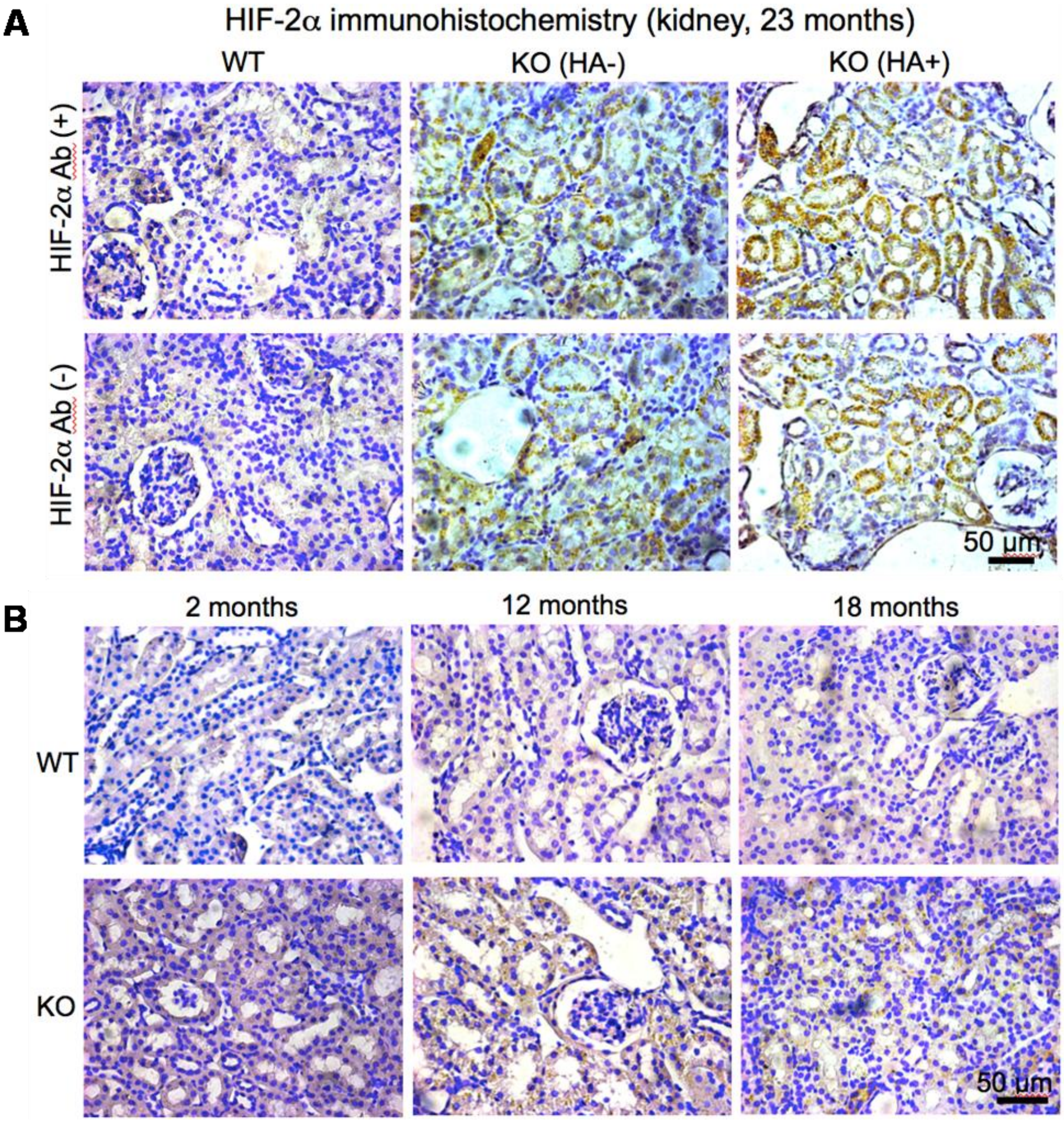

A
HIF-2α immunohistochemistry (kidney, 23 months)
WT
KO (HA-)
KO (HA+)
HIF-2α Ab (+)
HIF-2α Ab (-)
50 µm
B
2 months
12 months
18 months
WT
KO
50 µm

Supplementary Table S1. Relative *EPO* levels in the kidneys of 23-month mice

(*GAPDH* as control)

WT	0.3746612	0.6450844	0.7177532	0.9643506	1.277065	2.021026
KO (HA-)	0.1968609	0.5855199	1.017661	1.608839	2.362331	2.664026
KO (HA+)	0.7156684	1.817315	3.06013	3.466659	21.90904	79.12023

Supplementary Table S2. List of qPCR primers.

**Gene**	**Position**		**Primer sequences**
GAPDH	exon 4-5	Forward	AACTTTGGCATTGTGGAAGG
		Reverse	GGATGCAGGGATGATGTTCTG
EPO	exon 3-4	Forward	AGTCAACTTCTATGCTTGGA
		Reverse	CAGGATGGCTTCTGAGAG
VEGF	exon 2-3	Forward	ACGACAGAAGGAGAGCAGAAG
		Reverse	TCTCAATCGGACGGCAGTAG
LRRK2	exon 1-2	Forward	GGAGGCTCTGAAGAAGTTG
		Reverse	TACACTGGCAACTCTCATG
